# INvestigation on Routine Follow-up in CONgestive HearT FAilure Patients with Remotely Monitored Implanted Cardioverter Defibrillators SysTems (InContact)

**DOI:** 10.1186/s12872-018-0864-7

**Published:** 2018-06-28

**Authors:** Claudius Hansen, Christian Loges, Karlheinz Seidl, Frank Eberhardt, Herbert Tröster, Krum Petrov, Gerian Grönefeld, Peter Bramlage, Frank Birkenhauer, Christian Weiss

**Affiliations:** 1 Herz- und Gefäßzentrum am Krankenhaus Neu-Bethlehem, Humboldtallee 6, 37073 Göttingen, Germany; 2 SLK-Kliniken Heilbronn Klinikum am Plattenwald, Bad Friedrichshall, Germany; 3 Klinikum Ingolstadt, Ingolstadt, Germany; 4 Evangelisches Krankenhaus Kalk, Köln, Germany; 5 Marienhospital Stuttgart, Stuttgart, Germany; 6 Kreiskliniken Böblingen Standort Sindelfingen, Sindelfingen, Germany; 7 Asklepios Klinik Barmbek, Hamburg, Germany; 8 Institut für Pharmakologie und Präventive Medizin, Cloppenburg, Germany; 9 Abbott - St. Jude Medical GmbH, Eschborn, Germany; 10 Städtisches Klinikum Lüneburg gGmbH, Lüneburg, Germany

**Keywords:** Heart failure, Implantable cardioverter defibrillator, Cardiac resynchronisation therapy defibrillator, Remote monitoring, Packer heart failure clinical composite response

## Abstract

**Background:**

In heart failure (HF) patients with implantable cardioverter defibrillators (ICD) or cardiac resynchronisation therapy defibrillators (CRT-D), remote monitoring has been shown to result in at least non-inferior outcomes relative to in-clinic visits. We aimed to provide further evidence for this effect, and to assess whether adding telephone follow-ups to remote follow-ups influenced outcomes.

**Methods:**

InContact was a prospective, randomised, multicentre study. Subjects receiving quarterly automated follow-up only (telemetry group) were compared to those receiving personal physician contact. Personal contact patients were further divided into those receiving automated follow-up plus a telephone call (remote+phone subgroup) or in-clinic visits only.

**Results:**

Two hundred and ten patients underwent randomisation (telemetry *n* = 102; personal contact *n* = 108 [remote+phone: *n* = 53; visit: *n* = 55]). Baseline characteristics were comparable between groups and subgroups. Over 12 months, 34.8% of patients experienced deterioration of their Packer Clinical Composite Response, with no significant difference between the telemetry group and personal care (*p* > 0.999), remote+phone (*p* = 0.937) or visit (*p* = 0.940) patients; predefined non-inferiority criteria were met. Mortality rates (5.2% overall) were comparable between groups and subgroups (*p* = 0.832/*p* = 0.645), as were HF-hospitalisation rates (11.0% overall; *p* = 0.605/*p* = 0.851). The proportion of patients requiring ≥1 unscheduled follow-up was nominally higher in telemetry and remote+phone groups (42.2 and 45.3%) compared to the visit group (29.1%). Overall, ≥ 1 ICD therapy was delivered to 15.2% of patients.

**Conclusion:**

In HF patients with ICDs/CRT-Ds, quarterly remote follow-up only over 12 months was non-inferior to regular personal contact. Addition of quarterly telephone follow-ups to remote monitoring does not appear to offer any clinical advantage.

**Trial registration:**

clinicaltrials.gov: NCT01200381 (retrospectively registered on September 13th 2010).

## Background

Heart failure (HF) represents a substantial health burden, affecting approximately 26 million people on a global scale and accounting for 1–2% of all hospitalisations in Europe and America [1]. In patients with HF that cannot be effectively managed through medication alone, an implantable cardioverter defibrillator (ICD) or cardiac resynchronisation therapy defibrillator (CRT-D) is commonly indicated [2, 3].

Remote monitoring (RM) of implanted devices has been shown to have a number of advantages over exclusive in-clinic follow-up [4]. Firstly, it facilitates assessment of treatment regimens and optimisation of device functionality without the need for regular outpatient visits [4–6]. This reduces healthcare costs, issues surrounding appointment scheduling, and transportation costs for patients living in remote areas [7], and has been suggested to result in better adherence to follow-up [8]. Secondly, RM may encourage patients to take more responsibility for their own health status, and provide constant peace-of-mind that their device is functioning as intended [9, 10]. Indeed, RM can provide invaluable data for the early identification of clinically important irregularities, such as low battery output, device/lead faults, arrhythmias, and atrial fibrillation [11–14]. This has been demonstrated not only to allow timely prevention of clinical emergencies and mortality, but also to reduce the incidence of inappropriate device interventions [4, 6, 14–21]. Thus, the frequency of cardiovascular hospitalisation and long-term healthcare resource expenditure may be reduced [20, 22–24].

Given the available evidence, the Heart Rhythm Society (HRS) currently recommends that, where possible, use of remote care systems to monitor and interrogate implantable cardiac devices should be used in place of regular in-clinic visits, with a face-to-face evaluation taking place at least once every 12 months [11]. However, the value of supporting remote follow-ups with additional telephone contact has not yet been assessed.

In the present analysis, we aimed to provide more evidence that quarterly automated follow-ups are non-inferior to follow-ups which involve personal physician contact in HF patients with recently implanted ICD/CRT-D devices over 12 months. Secondly, we aimed to determine whether the type of physician contact affected outcomes. Of particular interest was whether the addition of quarterly physician telephone calls to remote follow-ups improved outcomes relative to both automated follow-ups only and traditional in-clinic visits.

## Methods

INvestigation on Routine Follow-up in CONgestive HearT FAilure Patients With Remotely Monitored Implanted Cardioverter Defibrillators SysTems (InContact) was a prospective, randomised, multicentre study conducted at 17 sites across Germany (see center list at end of article). The first patient was enrolled in February 2010 and study completion occurred in March 2014. The protocol was approved by the relevant ethics committees and the study was carried out in accordance with the Declaration of Helsinki 1964 and its amendments. All patients provided written informed consent.

### Patients and study groups

Only patients with ICD/CRT-D implantation indications consistent with guidelines (new device, generator replacement, or upgrade) were eligible for the present study. Additional inclusion criteria were: age ≥ 18 and < 80 years; ejection fraction ≤35%; New York Heart Association (NYHA) class I-III; and sufficient home infrastructure to support the use of a Merlin@home™ transmitter. Patients were excluded from the study if they had second-degree Mobitz type II or third-degree atrioventricular block; severe renal insufficiency; a life expectancy < 12 months; were pregnant; or were participating in a simultaneous study with an active therapy arm. Patients who had experienced a myocardial infarction or undergone a coronary angiology in the 3 months prior to enrolment were also excluded.

Following ICD/CRT-D implantation, patients were randomised in a 1:1 ratio into two study arms: those who were to receive quarterly personal contact with a physician (personal contact group) plus RM, and those who were to receive quarterly automated follow-up via Merlin.net only (telemetry group). Personal contact patients were further randomised into two subgroups: quarterly personal telephone calls with a physician/supporting nurse (remote+phone group), or quarterly in-clinic visits only (visit group). All of these groupings applied to the 12-month period between the first and thirteenth month after implantation. Regardless of study group, daily automatic alarm checks were activated for all patients throughout the study period.

### Study visits and documentation

A full medical history was taken for each patient prior to the day of ICD/CRT-D implantation (baseline). Prior to hospital discharge (PHD), quality of life (QoL) was assessed using the Minnesota Living with Heart Failure Questionnaire (MLHFQ; a self-administered, disease-specific questionnaire composed of 21 items, each with a 6-point scale [0 = no impact of HF on QoL, 5 = a great deal of impact]) [25]. The correct installation of the Merlin@home™ transmitter was verified within the first month post-intervention. Patients were provided with appropriate information on how to use their transmitter device. Quarterly scheduled sessions and device checks were set up by the clinic.

All patients attended an in-clinic visit 1 month (± 14 days) and 13 months (± 30 days) after ICD/CRT-D implantation, at which their Packer Heart Failure Clinical Composite Response score (Packer score) was determined, medication recorded, and QoL (MLHFQ) assessed. ICD/CRT-D function was then assessed for all patients at 4 and 7 months (± 14 days), either remotely or during the in-clinic appointment (visit group only). An optional follow-up was also carried out at 10 months. Adverse events and ICD/CRT-D measurements were recorded at each time point. Neither in-clinic or telephone interviews were pre-scripted, but were based around the discussion of device data.

### Outcome measures

The primary outcome measure was the proportion of patients with a worse Packer score at 13 months relative to their status at 1 month [26]. This composite outcome measure was determined via a stepwise assessment including the following parameters: HF-related death or hospitalisation (worse); deterioration of NYHA class or self-assessed health (worse); improved NHYA class or self-assessed health (improved); none of the above (unchanged).

Secondary outcome measures included the rates of all-cause mortality, HF-hospitalisations, and arrhythmias over the same 12-month period. The number of unscheduled (in-clinic, telephone-based or remote) follow-ups, the proportion of all follow-ups that had disease-relevant findings, and the number of delivered/appropriate ICD therapies were also documented. Changes in QoL between PHD, 1 and 13 months were assessed.

### Statistical analysis

The sample size calculation was based on a non-inferiority hypothesis, with a 15% margin for the occurrence of packer endpoint score deterioration at 13-month follow-up assumed. Pre-set values were 5% for the significance level and 80% for the power. A required sample size of 186 patients with complete datasets was calculated for a 1:1 (subgroups: 2:1:1) randomised design. After considering rates of drop out and incomplete data sets (predicted at approximately 20% overall), a total of 210 patients were planned for recruitment.

For continuous variables, a t-test, Wilcoxon signed-rank test or Mann-Whitney U-test were used for two-way comparisons and a Kruskal-Wallis test for three-way comparisons. A chi-square test (or Fisher’s exact test in the case of frequencies < 5%) was used for comparing categorical variables. In cases where more than two subgroups were compared and the comparison was significant, Pairwise comparisons of the groups were carried out and *p*-values were adjusted using the Bonferroni method. To test for the non-inferiority of telemetry compared to personal contact, the 95% confidence interval (CI) for the difference in the proportions of patients with a worsened Packer outcome at 13-month follow-up was calculated. Where the lower bound of this 95% CI exceeded − 0.15, automated follow-up was considered non-inferior to personal contact.

## Results

**Fig. 1:**
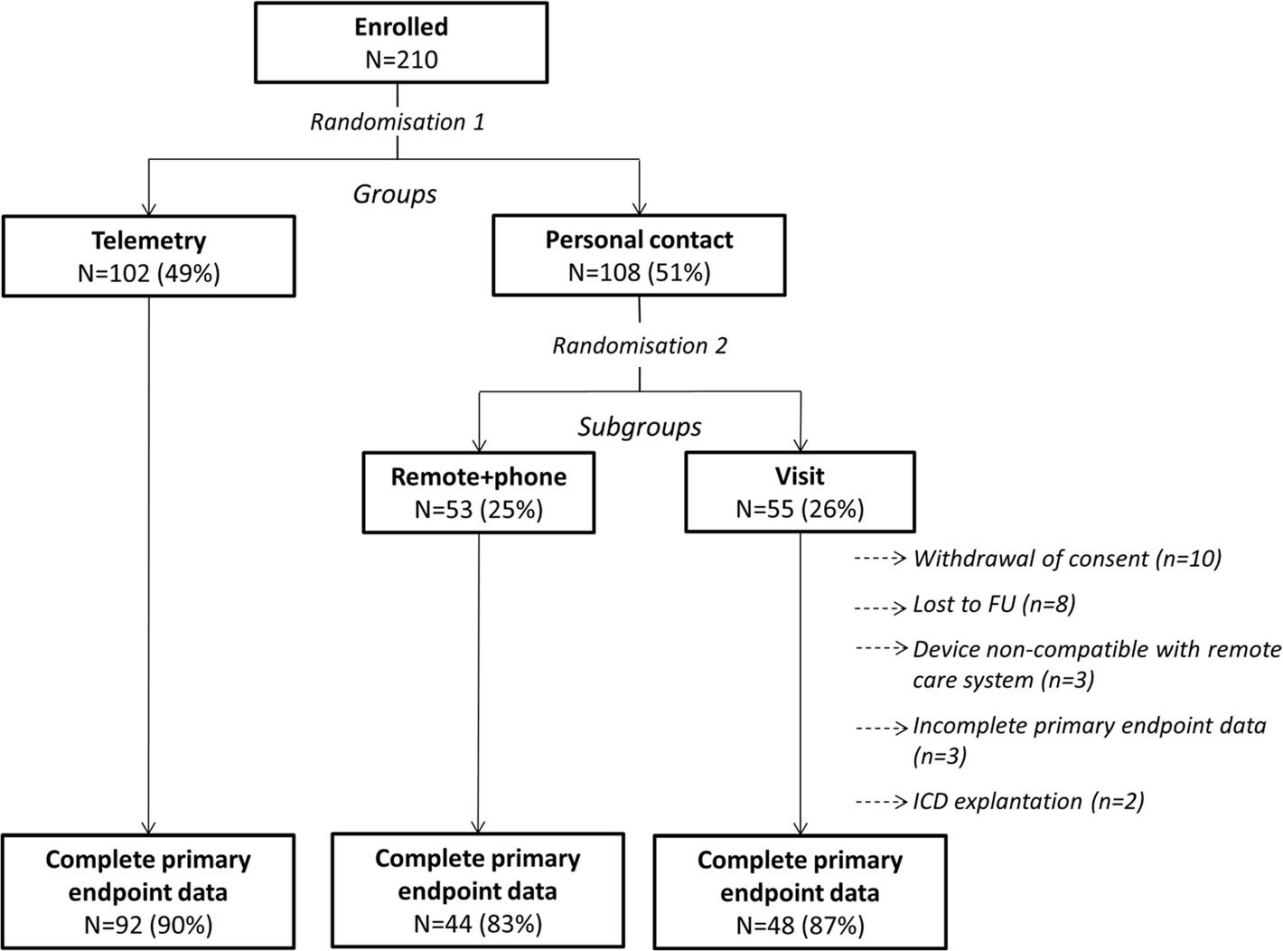
Study groups and patient flow. Legend: FU, follow-up. Reasons for device explantation (both remote+phone patients) were a floating structure at the atrial electrode and successful heart transplantation

The 210 patients initially enrolled in the study were randomised into either the telemetry (*n* = 102; 49%) or personal contact (*n* = 108; 51%) group (Fig. 1). Those in the personal contact group were further randomised into the remote+phone (*n* = 53; 25%) or visit (*n* = 55; 26%) subgroups.

### Baseline characteristics

Baseline characteristics were similar between all groups and subgroups (Table 1). Overall, patients had a mean age of 63.8 years, were predominantly male (84.3%), and had a mean NYHA class of 2.3 ± 0.7 [median 2; range 1–3; 42.9% NYHA III]. The majority had ischemic cardiac disease (59.0%).

**Table 1:** Baseline characteristics

	Telemetry	Personal contact			*p*-value (telemetry vs. personal contact)	*p*-value (telemetry vs. remote + phone vs. visit)
	(N = 102) n (%) / mean ± SD	All (*N* = 108) n (%) / mean ± SD	Remote + phone (*N* = 53) n (%) / mean ± SD	Visit (*N* = 55) n (%) / mean ± SD		
Age (years)	62.5 ± 12.2	65.1 ± 10.1	64.7 ± 9.1	65.4 ± 11.1	0.192	0.312
Female (%)	17 (16.7)	16 (14.8)	7 (13.2)	9 (16.4)	0.712	0.844
Disease parameters						
LVEF (%)	28.2 ± 7.1	28.3 ± 8.9	29.7 ± 10.8	26.9 ± 6.5	0.368	0.562
NYHA class (mean ± SD)	2.4 ± 0.6	2.3 ± 0.7	2.3 ± 0.7	2.3 ± 0.7	0.524	0.804
NYHA class (median)	2 (range 1–3)	2 (range 1–3)	2 (range 1–3)	2 (range 1–3)		
NYHA I	7.8	12.0	13.2	10.9		
NYHA II	48.0	46.3	43.4	49.1		
NYHA III	44.1	41.7	43.4	40.0		
Cardiac disease type					0.834	0.357
None (%)	0 (0.0)	1 (0.9)	1 (1.9)	0 (0.0)		
Ischemic (%)	58 (56.9)	66 (61.1)	30 (56.6)	36 (65.5)		
Non-ischemic (%)	41 (40.2)	38 (35.2)	19 (35.8)	19 (34.5)		
Other (%)	3 (2.9)	3 (2.8)	3 (5.7)	0 (0.0)		
ICD indication					0.861	0.508
Primary prevention	86 (84.3)	92 (85.2)	43 (81.1)	49 (89.1)		
Secondary prevention	16 (15.7)	16 (14.8)	10 (18.9)	6 (10.9)		
Cardiac medication						
None	5 (4.9)	9 (8.3)	4 (7.5)	5 (9.1)	0.319	0.578
Class 2 (beta-blockers)	96 (94.1)	99 (91.7)	49 (92.5)	50 (90.9)	0.491	0.751
Class 4	1 (1.0)	1 (0.9)	1 (1.9)	0 (0.0)	1.0	0.744
Amiodarone	12 (11.8)	12 (11.1)	5 (9.5)	7 (12.7)	0.882	0.856
Cardiac medication						
Diuretics	87 (85.3)	89 (82.4)	43 (81.1)	46 (83.6)	0.570	0.800
ACE inhibitors	81 (79.4)	86 (79.6)	38 (71.7)	48 (87.3)	0.969	0.134
ARB	18 (17.6)	16 (14.8)	10 (18.9)	6 (10.9)	0.578	0.456
Spironolactone	54 (53.5) ^a^	61 (56.5)	28 (52.8)	33 (60.0)	0.661	0.686
Device type					0.320	0.313
ICD Single Chamber	57 (55.9)	51 (47.2)	27 (50.9)	24 (43.6)		
ICD Dual Chamber	13 (12.7)	21 (19.4)	12 (22.6)	9 (16.4)		
CRT-D	32 (31.4)	36 (33.3)	14 (26.4)	22 (40.0)		
DF-4 connector	73 (71.6)	77 (71.3)	36 (67.9)	41 (74.5)	0.965	0.748
MLHFQ score PHD	33.6 ± 22.0	33.3 ± 22.0	33.7 ± 24.7	33.0 ± 19.3	0.861	0.976
MLHFQ score at 1 M	24.0 ± 20.3	21.8 ± 19.3	22.8 ± 23.7	20.9 ± 14.2	0.543	0.631

*LVEF* left ventricular ejection fraction, *NYHA* New York Heart Association, *ICD* implantable cardioverter-defibrillator, *ACE* angiotensin-converting enzyme, *ARB* angiotensin II receptor blockers, *CRT-D* cardiac resynchronization therapy implantable cardioverter-defibrillator, *MLHFQ* Minnesota Living with Heart Failure Questionnaire, *PHD* pre-hospital discharge, *1 M* 1 month

^a^ Data was missing for 1 patient

Beta-blockers were the most commonly prescribed cardiac drug at baseline (92.9%), followed by amiodarone (11.4%). Only 6.7% of patients were not receiving any HF medication. Diuretics were being taken by 83.8% of the study population, angiotensin-converting enzyme inhibitors by 79.5%, spironolactone by 54.8%, and angiotensin II receptor blockers by 16.2%.

The majority of patients received their ICD in a primary prevention setting (84.8%). The most frequently implanted device was a single chamber ICD (51.4%), followed by a CRT-D (32.4%) and dual-chamber ICD (16.2%). A DF-4 connecter was implanted in 71.4% of cases. The most common ICD model was Fortify (36.2%) followed by Current + (18.1%) and Unify (12.4%). All other models were used at a frequency of < 7%.

### Outcomes at 13 months

Of the patients enrolled, 184 were available for the primary endpoint analysis, 92 (90.2%) in the telemetry and 92 (85.2%) in the personal contact group (remote+phone: 44 patients, 83.0%; visit: 48 patients, 87.3%).

#### Primary endpoint: Packer heart failure clinical composite response

**Fig. 2:**
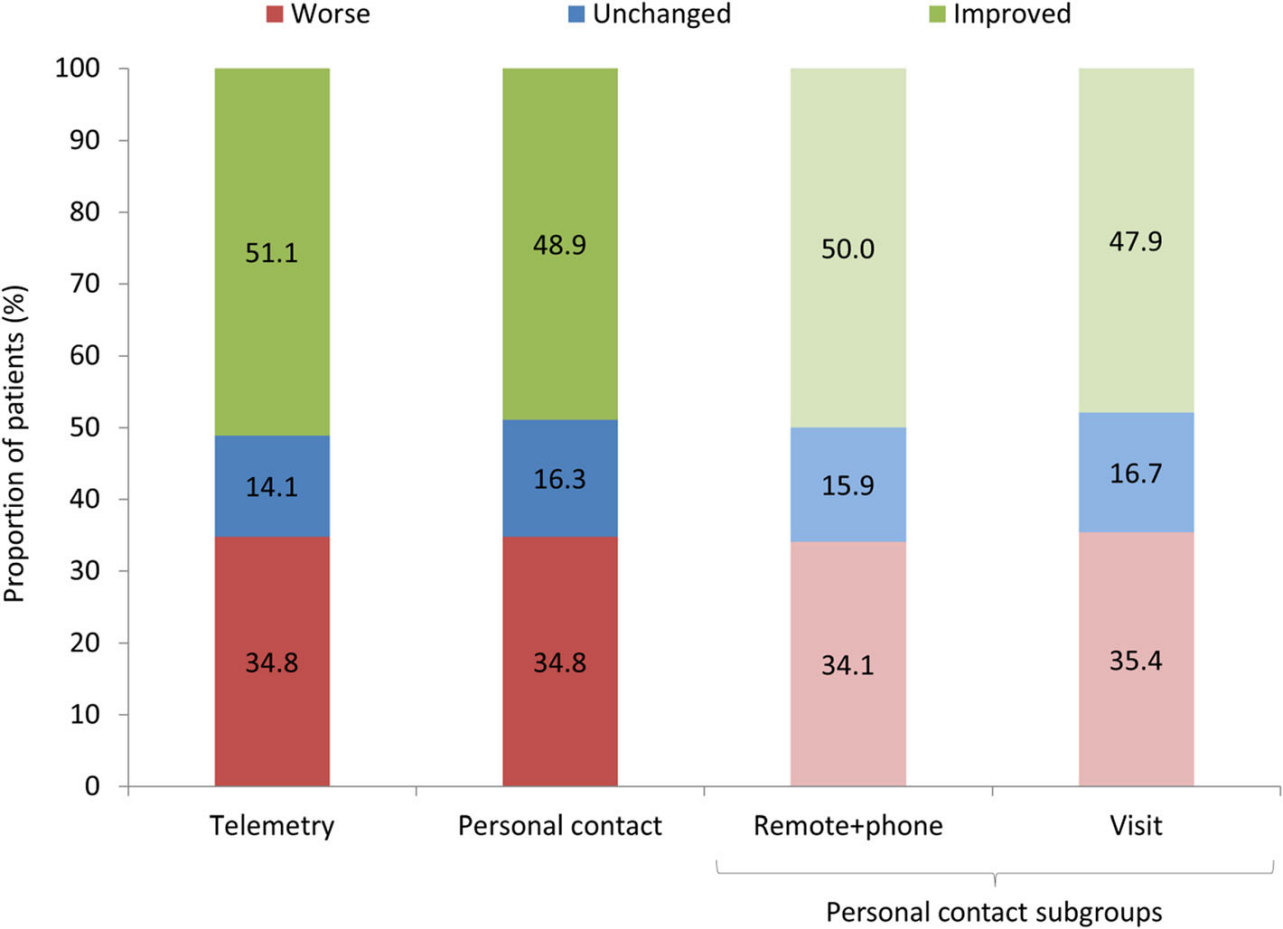
Change in Packer score at 13 months vs. 1 month. Legend: Proportions are relative to the number of patients in each group/subgroup with all relevant data available (telemetry: *n* = 92; personal contact: n = 92 including remote+phone: *n* = 44 and visit: *n* = 48). The distribution of the type of change in Packer score (worse, unchanged or improved) was not significantly different between telemetry vs. personal care (*p* = 0.855) or telemetry vs. remote+phone vs. visit groups/subgroups (*p* = 0.967)

Overall, 34.8% of patients had a worse Packer score at 13 months relative to 1 month, with 15.2% remaining unchanged and 50.0% experiencing an improvement. This distribution was not significantly different between telemetry and personal care group (*p* = 0.855) or telemetry, remote+phone and visit groups/subgroups (*p* = 0.967) (Fig. 2**).**

When considering only the proportion of patients with a worse packer score, no significant difference was detected between the telemetry compared to personal care group (*p* > 0.999). As the difference between means was 0.000 and the 95% CI (− 1.1376 to 0.1376) lower limit greater than the predefined non-inferiority delta (− 0.15), non-inferiority of telemetry compared to personal follow up was concluded. Furthermore, no significant difference was found when comparing the telemetry group to the remote+phone subgroup (*p* = 0.937), the telemetry group to the visit subgroup (*p* = 0.940), or the remote+phone and visit subgroups (*p* = 0.894).

#### Mortality and HF-hospitalisation

During the course of the study, mortality rates were comparable between telemetry and personal contact groups (4.9% vs. 5.6%, *p* = 0.832), and telemetry, remote+phone and visit groups (4.9% vs. 7.5% vs. 3.6%, respectively; *p* = 0.645) (Table 2). Of the 11 deaths which occurred during the course of the study, 4 were from cardiac origin (HF and coronary artery disease), 4 were from non-cardiac origin, and 3 were of “unclear” origin. The origin of death was “unclear” in three of five patients (60%) in the telemetry group compared to none of six (0%) in either of the personal contact groups.

**Table 2:** Secondary outcomes: Packer sub-items, cardiac parameters, and QoL

	Telemetry (*N* = 102) n (%) / mean ± SD	Personal contact			p-value (telemetry vs. personal contact)	p-value (telemetry vs. remote + phone vs. visit)
		All (*N* = 108) n (%) / mean ± SD	Remote + phone (N = 53) n (%) / mean ± SD	Visit (N = 55) n (%) / mean ± SD		
Packer sub-items						
Death	5 (4.9)	6 (5.6)	4 (7.5)	2 (3.6)	0.832	0.645
Cardiac	1 (20.0)	3 (50.0)	2 (50.0)	1 (50.0)	0.259	0.292
Non-cardiac	1 (20.0)	3 (50.0)	2 (50.0)	1 (50.0)		
Origin unclear	3 (60.0)	0 (0.0)	0 (0.0)	0 (0.0)		
HF-hospitalisation^b^	10 (9.8)	13 (12.0)	6 (11.3)	7 (12.7)	0.605	0.851
NYHA class at 13 M vs. 1 M^a^					0.639	0.715
Worse	22 (25.3)	19 (22.6)	7 (17.9)	12 (26.7)		
Unchanged	49 (56.3)	53 (63.1)	27 (69.2)	26 (57.8)		
Improved	16 (18.4)	12 (14.3)	5 (12.8)	7 (15.6)		
Change in NYHA class at 13 M vs. 1 M^a^	−0.07 ± 0.71	−0.10 ± 0.63	−0.8 ± 0.62	−0.11 ± 0.65	0.888	0.912
Self-assessment^c^						
Worse	8 (9.2)	10 (11.8)	6 (15.0)	4 (8.9)	0.582	0.564
Unchanged	24 (27.6)	21 (24.7)	9 (22.5)	12 (26.7)		
Improved	55 (63.2)	54 (63.5)	25 (62.5)	29 (64.4)	0.966	0.982
Cardiac events						
Stored tachycardia^d^	25 (24.5) / 4.8 ± 15.6	23 (21.3) / 2.2 ± 9.0	11 (20.8) / 1.0 ± 4.5	12 (21.8) / 3.5 ± 11.8	0.579 / 0.431	0.850 / 0.680
VT/VF^d^	20 (19.6) 2.1 ± 9.1	17 (15.7) 1.3 ± 6.7	8 (15.1) 0.8 ± 3.7	9 (16.4) 1.8 ± 8.7	0.462 / 0.409	0.752 0.689
SVT^d^	18 (17.6) 2.8 ± 11.4	11 (10.2) 1.0 ± 5.4	4 (7.5) 0.2 ± 1.0	7 (12.7) 1.7 ± 7.5	0.117 0.097	0.216 0.180
Cardiac decompensations	9 (8.8)	9 (8.3)	5 (9.4)	4 (7.3)	0.899	0.915
QoL						
Change in MLHFQ score at 13 M vs. PHD	−8.4 ± 20.3	−10.5 ± 21.6	−12.1 ± 22.7	−9.1 ± 20.8	0.472	0.724
Change in MLHFQ score at 13 M vs. 1 M	0.7 ± 16.8	2.7 ± 20.5	3.0 ± 22.2	2.4 ± 19.1	0.666	0.837

*NYHA* New York Heart Association, *13 M* 13 months, *HF* heart failure, *VF* ventricular fibrillation, *VT* ventricular tachycardia, *SVT* supraventricular tachycardia, *MLHFQ* Minnesota Living with Heart Failure Questionnaire, *PHD* pre-hospital discharge, *1 M* 1 month

^a^Data based on non-missing values (*n* = 171)

^b^Data based on non-missing values (*n* = 174)

^c^Data based on non-missing values (*n* = 172)

^d^Percentage of patients / number of tachycardia events

HF-hospitalisation occurred at similar rates in the telemetry and personal contact groups (9.8% vs. 12.0%, *p* = 0.605) (Table 2). This was also the case when comparing telemetry, remote+phone and visit groups/subgroups (9.8% vs. 11.3% vs. 12.7%, *p* = 0.851).

#### Follow-ups

Overall, 100 patients (47.6%) had at least one relevant finding from at least one ICD/CRT-D follow-up, with no significant differences between the telemetry and personal contact groups (*p* = 0.693) or telemetry, remote+phone and visit groups/subgroups (*p* = 0.789) (Table 3). Again, findings were predominantly medical (82 patients; 39.0% overall), with only 35 patients found to have ICD-related technical findings (16.7% overall). This trend was consistent across all groups and subgroups.

**Table 3:** Secondary outcomes: follow-ups between months 1 and 13

	Telemetry (N = 102) n (%) / mean ± SD	Personal contact			p-value (telemetry vs. personal contact)	p-value (telemetry vs. remote+phone vs. visit)
		All (N = 108) n (%) / mean ± SD	Remote+phone (N = 53) n (%) / mean ± SD	Visit (N = 55) n (%) / mean ± SD		
FU duration (days)	372.8 ± 100.3	366.9 ± 127.5	349.9 ± 147.8	384.0 ± 102.0	0.689	0.490
Unscheduled FUs						
No. of FUs /patient	1.2 ± 2.6	0.9 ± 1.8	1.0 ± 1.7	0.8 ± 2.0	0.550	0.285
Total number	120	99	54	45		
Considered reasonable	90 (75.0)	77 (77.8%)	43 (79.6)	34 (75.6)	0.631	0.797
With findings	55 (45.8)	57 (57.6)	28 (51.9)	29 (64.4)	0.084	0.104
Medical	40 (33.3)	37 (37.4)	17 (31.5)	20 (44.4)	0.534	0.335
Technical	9 (7.5)	12 (12.1)	9 (16.7)	3 (6.7)	0.249	0.126
All FUs						
Patients with relevant findings at ≥1 FU	50 (49.0)	50 (46.3)	26 (49.1)	24 (43.6)	0.693	0.789
Medical	42 (41.2)	40 (37.0)	19 (35.8)	21 (38.2)	0.539	0.803
Technical	16 (15.7)	19 (17.6)	11 (20.8)	8 (14.5)	0.711	0.642
Patients with ICD therapy at ≥1 FU	17 (16.7)	15 (13.9)	7 (13.2)	8 (14.5)	0.576	0.839
ICD shock	9 (8.8)	9 (8.3)	4 (7.5)	5 (9.1)	0.899	0.792
ATP therapy	16 (15.7)	14 (13.0)	7 (13.2)	7 (12.7)	0.573	0.851
System revisions						
Patients requiring ≥1	6 (5.9)	1 (0.9)	1 (1.9)	0 (0)	0.059	0.141
Mean per patient	0.06 ± 0.02	0.01 ± 0.10	0.02 ± 0.14	0.00 ± 0.00	0.046	0.118

*FU* follow-up, *ICD* implanted cardioverter-defibrillator, *ATP* anti-tachycardia pacing

**Fig. 3:**
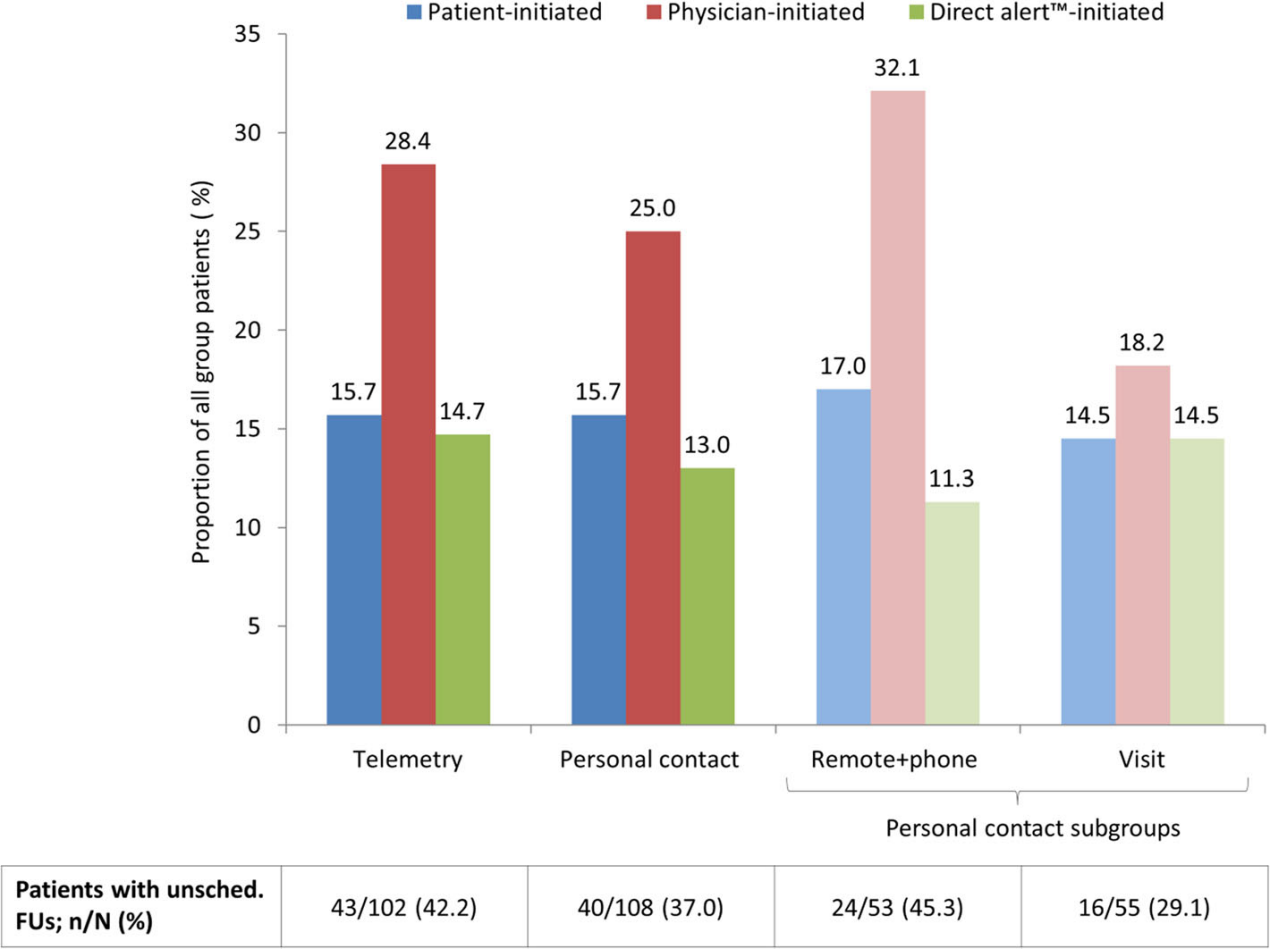
Unscheduled follow-ups between 1 and 13 months. Legend: unsched., unscheduled; FU, follow-up. The percentages displayed in the table and graph are proportional to the total number of patients within the respective group/subgroup that had ≥1 unscheduled follow-up. The three initiation types are not exclusive and any one patient may be represented by multiple bars. The distribution of initiation types was not significantly different between telemetry vs. personal care or telemetry vs. remote+phone vs. visit groups/subgroups. Follow-ups could be in-house or remote

In total, 219 unscheduled follow-ups (102 in-clinic; 106 remote; 11 telephone-based) occurred in 83 patients over a mean period of 369.8 ± 114.7 days. The proportion of patients with at least one unscheduled follow-up was nominally higher in the telemetry compared to personal contact group (42.2% vs. 37.0%, *p* = 0.448), and in the telemetry and remote+phone groups (42.2 and 45.3%) compared to the visit group (29.1%, *p* = 0.171) (Fig. 3). The proportion of patients with physician-initiated unscheduled follow-ups was also nominally higher in telemetry and remote+phone groups (28.4 and 32.1% vs. 18.2%), though did not reach statistical significance. The unscheduled follow-up was considered reasonable in 75.0 and 77.8% of cases in the telemetry and personal contact groups (*p* = 0.631), and 79.6 and 75.6% of remote+phone and visit subgroups (*p* = 0.797 also vs. telemetry group) (Table 3). However, only 51.1% yielded findings, with this proportion nominally higher in the visit subgroup (64.4%) compared to the telemetry (45.8%) and remote+phone (51.9%) group/subgroup (*p* = 0.104). Findings of a medical nature were generally more common than technical findings (35.2% vs. 9.6%, overall). System revisions were required in six patients (5.9%) in the telemetry group (three dislodgements, one myocardial perforation, one planned placement of an epicardial left ventricular lead, and one anticipated perforation of device through skin) and one patient (0.9%) in the personal contact (remote+phone) group (dislodgement and myocardial perforation; *p* = 0.059) (Table 3).

#### Arrhythmias and ICD therapies

Stored tachycardias occurred in 22.9% of the population, with no significant differences between groups (*p* = 0.579) or subgroups (*p* = 0.899) (Table 2). Rates of ventricular fibrillation/tachycardia and supraventricular tachycardia (SVT) were statistically comparable between telemetry and personal contact groups (19.6% vs. 15.7%, *p* = 0.462; and 17.6% vs. 10.2%, *p* = 0.117, respectively). The same was true for subgroup comparisons.

Overall, at least one ICD therapy was delivered to 32 patients (15.2%) during follow-up, with ATP therapy given to 14.3% and shock to 8.6% of all patients. These trends were consistent across groups and subgroups.

#### Functional status and quality of life

Overall, NYHA class worsened by a mean of 0.08 ± 0.672 between 1 and 13 months (median 2, range 1–3), with no significant differences found for group (*p* = 0.888) or subgroup (*p* = 0.912) comparisons (Table 2). NYHA class worsened in 24.0%, was unchanged in 59.6% and improved in 16.4% of the total population, again without significant differences in distribution between groups (*p* = 0.639) or subgroups (*p* = 0.715).

The overall mean change in MLHFQ score between PHD and 13 months was − 9.38, with a mean increment of 1.64 between 1 and 13 months. This trend was consistent across all groups and subgroups, with no significant differences detected (Table 2).

## Discussion

We aimed to compare the outcomes of HF patients receiving quarterly automated ICD/CRT-D follow-up compared to those receiving personal physician follow-up at the same intervals over 12 months. We further explored the impact of the type of physician contact on outcomes. Given its prior absence in the literature, the effect of adding telephone follow-up to remote follow-up was of particular interest. We found that comparable proportions of the different follow-up groups/subgroups experienced a worsening of their Packer score over the course of the study. The same was true for rates of mortality, HF-hospitalisation, unscheduled follow-ups, and inappropriate ICD therapy. Taking into account the robust comparability of baseline characteristics, our data suggest that a 12-month follow-up programme via a fully automated system is non-inferior to a programme involving regular physician contact. Furthermore, the addition of telephone follow-ups to quarterly automated follow-ups appears not to produce a clinical advantage, though further corroboratory data would be instructive.

### Packer heart failure clinical composite response

Change in Packer score was used as a primary assessment criterion as it is considered to provide a clinically meaningful overview of HF status, encompassing changes in both disease parameters and risk of major clinical events [26, 27]. However, it has been employed in only a few RM studies to date. The 664-patient IN-TIME study used this endpoint to demonstrate that telemonitoring plus standard care resulted in 8.3% fewer patients deteriorating over 12 months compared to those receiving standard care only (*p* = 0.013) [16]. While this did not address the question of whether remote follow-up alone can replace physician follow-up, it did suggest the advantage of combining RM plus personal contact over exclusive face-to-face assessment.

In terms of studies assessing the value of entirely remote follow-ups, only the 16-month EVOLVO study appears to have employed the Packer composite endpoint [28]. This study compared 99 patients exclusively receiving remote follow-up with 101 receiving in-clinic follow-up only, and found no significant difference in the proportion of patients with a worse Packer score at 16 months relative to baseline (34% vs. 44%, respectively). Concurrently, 35% of all patients in the present study were found to have experienced a deterioration in Packer score between 1 and 13 months, with this applying to a comparable proportion of telemetry and personal contact (including visit) patients. Furthermore, the non-inferiority of the entirely remote approach was statistically indicated according to the predefined criteria. Thus, our data support previous findings that automated follow-up regimes are non-inferior to in-clinic follow-up regimes in terms of composite response. In addition, our data go further, indicating for the first time that addition of a telephone call to quarterly remote follow-ups may not provide any significant advantage over RM alone. In light of this, current HRS guidelines appear to be appropriate [11], though further validation of our findings in larger-scale studies is required.

### Mortality

There was no difference in all-cause mortality at 12 months in the telemetry compared to the visit group (4.9% vs. 3.6%, respectively). This is in line with other randomised control trials (RCTs), such as the MORE-CARE (9.2% vs. 7.9%, *p* = 0.594) and TELECART (7.9% vs. 8.9%, *p* = 0.54) studies and a large-scale meta-analysis comparing RM to outpatient follow-up (odds ratio [OR] 0.83, 95% CI 0.58–1.17, *p* = 0.285) [17]. Conversely, several observational studies have identified lower mortality rates in patients receiving telemonitoring compared to those followed up in-house [15, 18]. Perhaps the most convincing of these is ALTITUDE, which compared 10,272 matched ICD and CRT-D patients with or without remote network follow-up over 5 years post-implant [15]. The risk of death was found to be halved by application of RM (ICD: hazard ratio [HR] 0.56, 95% CI 0.47–0.67, *p* < 0.001; CRT-D; HR 0.45, 95% CI 0.39–0.53, p < 0.001); however, follow-up modality was decided by the treating physician based on device availability and logistics, introducing a probable bias. Furthermore, patients may receive more rigorous assessment in the context of a RCT, resulting in a higher quality of in-house care provision. Regardless of the cause, it is important to note that findings from RCTs, however robust, may not directly translate into a real-world context. This should be taken into consideration when reflecting upon the finding that there was no significant difference in mortality rates between telemetry and remote+phone groups, even when stratified by cause of death. While our analysis was explorative, this once more indicates that addition of telephone contact to remote follow-up may not be advantageous for reducing fatalities, at least in the context of an RCT. Of interest is the finding that the cause of death was “unclear” in 60% of telemetry patients compared to none of the personal contact patients, suggesting that the availability of certain information may be lost through entirely remote management. Nonetheless, further investigation of the present findings through real-world observational registries may be informative.

### HF-hospitalisations

No significant differences were found between groups or subgroups with respect to the frequency of HF-hospitalisations. Again, this was consistent with previous findings from RCTs, including CONNECT and EVOLVO [14, 28–30]. These studies reported annual rates ranging from 0.39–0.50 cardiovascular hospitalisations per patient, with no difference between RM and in-clinic groups (*p* = 0.99 and *p* = 0.464 for each study, respectively) [14, 28]. Furthermore, a recently published meta-analysis pooling data from 7 RCTs found no significant reduction in the odds of hospitalisation with RM compared to standard care (OR 0.83; 95% CI 0.63–1.10; *p* = 0.196) [17]; however, it should be noted that this referred to hospitalisation for all-causes. Nonetheless, the apparent lack of impact of telephone contact in addition to remote follow-up on HF-hospitalisation rates in the present study is unsurprising.

Interestingly, observational studies have consistently associated telemetry with a reduction in hospitalisation frequency [12, 23, 24]. Again, this discrepancy is likely due to socioeconomic and geographical bias reflective of real-world trends and applications. Thus, evaluating the effect of RM plus telephone follow-up on HF-hospitalisation rates in a real-world setting may prove interesting. Of note, observational studies have also linked RM to a shorter duration of hospitalisation episodes and lower associated costs [12, 23, 24], with one analysis specifically identifying a reduction in cardiovascular hospitalisation expenditure as the key driver for the cost savings associated with RM [23]. Though these parameters were beyond the scope of the present analysis, they should be borne in mind for future studies evaluating the value of telephone contact as an adjunct to RM.

### Unscheduled follow-ups

Though not reaching statistical significance, the proportion of patients who had at least one unscheduled follow-up in the present study was approximately 15% higher in the telemetry and remote+phone groups compared to the visit group. This appears to have been largely driven by more frequent physician-initiated follow-ups. A higher yearly rate of additional follow-ups in remotely monitored patients compared to those regularly attending in-clinic appointments was also noted in TRUST (0.78 vs. 0.50 visits/person/year, *p* = 0.009), CONNECT (2.24 vs. 1.95 visits/person/year, *p* = 0.099), EuroEco (0.95 vs. 0.62 visits/person/year, *p* = 0.005), and MORE-CARE (IRR 2.80, 95% CI 2.16–3.63, *p* < 0.001) studies [13, 14, 29, 30]. In addition, a recent meta-analysis reported RM to be associated with a nonsignificant trend towards greater odds of experiencing an unscheduled follow-up (OR 1.29; 95% CI 0.99–1.67, *p* = 0.061) [17]. This trend may be representative of physicians feeling less confident about their patient’s conditions when not meeting them on a regular basis and thus being more likely to initiate unscheduled contact. However, such contact was not necessarily required in all cases, with findings at an unscheduled follow-up nominally less frequent in the telemetry and remote+phone groups compared to the visit group. Given that remote+phone and telemetry patients cited “perceived clinical symptoms” as the reason for patient-initiated unscheduled follow-up significantly more frequently than visit patients (16.7 and 5.8% vs. 2.2%, *p* = 0.002), insecurity in those unable to meet face-to-face with their treating physician on a regular basis may also have contributed. Taken together, our data may indicate that neither telemetry nor telephone contact can replace in-clinic visits for instilling confidence in patients and physicians alike, though the present study was not specifically designed to identify such an effect.

System revisions were more common in the telemetry compared to the personal contact group. A similar trend was reported by the TRUST trial, with home monitoring found to result in a greater rate of in-person system revisions compared to conventional monitoring (29.9% vs. 14.5% at 100 days; *p* = 0.018) [31, 32]. While TRUST authors attribute this finding to the earlier detection of technical issues using RM, this explanation is less applicable to the present study, given that RM and direct alerts were activated for all patients. Thus, the reason for this imbalance remains illusive and merits further exploration.

### Arrhythmias and ICD therapies

There were no significant differences between groups and subgroups with respect to cardiac decompensations or number of stored tachycardias. Similarly, a study by Marcantoni et al. designed particularly to explore the effect of RM on supraventricular and ventricular arrhythmias also found no difference in incidence when comparing patients followed remotely to those followed in the clinic [19]. However, the study did report a reduction in associated events in the RM group, explained as due to the earlier detection and pre-emptive treatment of such arrhythmias.

ICD therapy (shock or ATP) is associated with an increased risk of mortality and HF deterioration, regardless of its appropriacy [33, 34]. This is thought to be partly due to the negative inotropic consequences of the therapy, which may result in a significant reduction in cardiac index [35–37]. Consequently, avoidance of inappropriate therapy is paramount. In the present study, approximately 15% of patients received ICD therapy over 12 months, with no notable differences across groups or subgroups. This is consistent with the majority of previously published studies [6, 15, 17, 21].

### Quality of life

It has been suggested that RM can reduce the inconvenience of attending in-clinic visits and anxiety between follow-ups, resulting in a positive effect on QoL [10]. However, most studies have found it to have no effect on QoL [5, 29], with the exception of the EVOLVO study [28]. Although MLHFQ score fell by approximately 9 points between PHD and 13 months in the present study, indicating an overall improvement in QoL, a minor deterioration between months 1 and 13 occurred in all groups. This deterioration was marginally smaller in the remote+phone group, though this effect was not significant due to the large within-group variations (demonstrated by very large standard deviations). Our data are thus in line with previous evidence that neither automated remote follow-up nor personal contact appear to be superior for improving MLHFQ-measured patient QoL.

### Limitations

This was a prospective, randomised multicentre study in Germany with patients being monitored using the Merlin@home™ system. We regard this, together with the possible use of different ICD/CRT-D devices, as a particular strength of the study. However, our findings may not be generalizable to patients using other RM systems. In addition, while we endeavoured to ensure consecutive enrolment, this was not always possible; as such, we cannot guarantee the elimination of selection bias. Furthermore, the relevance of medical and technical findings was determined at the physician’s discretion, potentially leading to subjectivity bias. Nevertheless, randomisation ensured that any one physician was treating both RM and personal contact patients, likely resulting in a reasonably uniform impact of such bias across groups and subgroups. Finally, limited patient numbers may have prevented detection of small but significant effects owing to insufficient statistical power. Indeed, initial sample size calculations were performed based on between-group comparisons, with subgroup comparisons thus being suboptimally powered. As such, large-scale observational studies would be informative.

## Conclusions

In HF patients with recently implanted ICDs/CRT-Ds, entirely automated remote follow-up is non-inferior to follow-up with personal contact over a period of 12 months. Furthermore, the addition of a telephone call to quarterly automated follow-ups does not appear to be beneficial for improving outcomes, though further studies are necessary to corroborate this finding. Nevertheless, the present study provides additional evidence in support of the safe and effective replacement of regular physician contact with fully-automated follow-up in this patient population.

## Center list - Germany

Herz-und Gefäßzentrum am Krankenhaus Neu-Bethlehem Göttingen gGmbH, 37,073 Göttingen; Städtisches Klinikum Lüneburg gGmbH, 21,339 Lüneburg; SLK-Kliniken Heilbronn GmbH Klinikum am Plattenwald, 74,177 Bad Friedrichshall; Klinikum Ingolstadt GmbH, 85,049 Ingolstadt; Evangelisches Krankenhaus Kalk, 51,103 Köln; Marienhospital Stuttgart, 70,199 Stuttgart; Klinikum Sindelfingen-Böblingen gGmbH, 71,065 Sindelfinden; Asklepios Klinik Barmbek, 22,307 Hamburg; Kardiologische Gemeinschaftspraxis am Park Sanssouci, 14,471 Potsdam; Segeberger Kliniken GmbH, 23,795 Bad Segeberg; Kardiologische Praxis – Partnergesellschaft, 71,634 Ludwigsburg; Asklepios Klinik Bad Oldesloe, 23,843 Bad Oldesloe; Evangelisches Krankenhaus Bielefeld gGmbH, 33,617 Bielefeld; Marienhaus Klinikum St. Elisabeth-Krankenhaus, 56,564 Neuwied; Asklepios Klinik St. Georg, 20,099 Hamburg; Universitätsklinikum Essen (AöR), 45,122 Essen; Praxis Dr. med. Balbach / Ruppert, 72,622 Nürtingen.

## Abbreviations

ACE: Angiotensin converting enzyme
ARB: Angiotensin receptor blocker
ATP: Antitachycardia pacing
CI: Confidence interval
CRT-D: Cardiac resynchronisation therapy defibrillator
FU: Follow up
HF: Heart failure
HRS: Heart Rhythm Society
ICD: Implantable cardioverter defibrillator
LVEF: Left ventricular ejection fraction
MLHFQ: Minnesota Living with Heart Failure Questionnaire
NYHA: New York Heart Association
PHD: Pre-hospital discharge
QoL: Quality of life
RM: Remote monitoring
SD: Standard deviation
SVT: Supraventricular tachycardia
VF: Ventricular fibrillation
VT: Ventricular tachycardia
